# Supplementary material for: Structural insights into target detection by the Serratia type III CRISPR complex and its deployment in SNP identifications
Source: J Biol Chem. 2026 Sep 23;302(11):113595. doi: 10.1016/j.jbc.2026.113595 (PMC13737744; doi:10.1016/j.jbc.2026.113595)
Supplement: Supplementary file 1 [file mmc2.docx]

Structural insights into target detection by the *Serratia* type III CRISPR complex and its deployment in SNP identification

Calvin C. Perdigao^1^, Luqman O. Ajisafe^1^, Anju T. Sunny^1^, Si Wu^1^, Terje Dokland^2^ and Jack A. Dunkle^1^*

^1^ Department of Chemistry and Biochemistry, University of Alabama, Tuscaloosa, AL, USA

^2^ Department of Microbiology, University of Alabama at Birmingham, Birmingham, AL, USA

*Corresponding author: Jack A. Dunkle

E-mail: [jadunkle@ua.edu](mailto:jadunkle@ua.edu)

**Supporting Information**

**Figure S1. Plasmid map for pACYC-Csm.** Key features of the pACYC-Csm plasmid, such as open reading frames for the CRISPR system and the antibiotic resistance gene, are annotated.

**Figure S2. Plasmid map for pTarget.** Key features of pTarget are annotated such as the antibiotic resistance gene 'Beta Lactamase' and the location that encodes the target RNA 'dotA'.

**Table S1. Plasmid sequences, gene fragments and oligos used in the study.**

| Name | Sequence (5’-3’) | |
| --- | --- | --- |
| Plasmids | | |
| pACYC-Csm | 1 atgcacgaac cccccgttca gtccgaccgc tgcgccttat ccggtaacta tcgtcttgag  61 tccaacccgg aaagacatgc aaaagcacca ctggcagcag ccactggtaa ttgatttaga  121 ggagttagtc ttgaagtcat gcgccggtta aggctaaact gaaaggacaa gttttggtga  181 ctgcgctcct ccaagccagt tacctcggtt caaagagttg gtagctcaga gaaccttcga  241 aaaaccgccc tgcaaggcgg ttttttcgtt ttcagagcaa gagattacgc gcagaccaaa  301 acgatctcaa gaagatcatc ttattaatca gataaaatat ttctagattt cagtgcaatt  361 tatctcttca aatgtagcac ctgaagtcag ccccatacga tataagttgt aattctcatg  421 tttgacagcc ttcagatccg atagactagc cgctggtaat aatacgactc actataggga  481 gagaattcta agaccgaaag tcggaaacaa agaggattta tatgacatgg gtttgccatc  541 tggcgcgtta ccgcctctgt tttcaaagca tcgaaccgct ggcgctgccg ctgtttagcg  601 gctccatgct gcgcggcgcg ttcggccatg cgctgcggcg catctgttgc atcagccgtc  661 agaaacagtg tgacggctgc ccgttgctgg ccggttgcca gtacccgctg ttgtttgagc  721 cgcgcttgct gcaaaacacc cccggccagc cccaaccggc tccgccttat gtgctggagc  781 cagcggccgt tcagagggag cttgccgcag gggaaatctg gccggtagac gtggtgctgc  841 acggcccggc gctaccgcac ctgagcctga ttattctggc ctggatgcag gcagctgttc  901 aggggtttgg cagccagcgg gtgccagcac agttgtgcca ggtgcaggta gagcaacccg  961 atactgaagc gcgctggctg acgatctggc gtcacgacca gccgtttatc ctgccacacg  1021 ccaccacgca aacaccgcca ccgcccccgg cggcagaggc gctgcgcctg cacttcacca  1081 ccccgacccg gctgttgcgc caggggcaac tggtacgggt gcgtgaactt caggcacacg  1141 acctgctggc cgcgctggag cgacgcctgc acacgctggc cgcctcgctg ggcattgccc  1201 cgccgccgtc gctgaccacg caactcgtta cgctgcaacc cggccagttg cgctggatga  1261 actggcaacg ctactccagc cgccagcaac aggagatgaa tctcggcgga tttatcggtg  1321 acgttacgct ccacggcgag ctgacgccgc tgtggacatg gctgtggctg ggccagtggt  1381 tgcatgtggg caaaaacagc agcttcgggc tgggacgcta tcagttagag acggttcccc  1441 gctaaaagct ttcctgtgag cagcgaaagc ctagcataac cccttggggc ctctaaacgg  1501 gtcttgaggg gttttttgtt atacgcgaga taatcacttg catagctgcg tatggaggaa  1561 gcaactcttg agtgttaata tgttgacccc tgtattaggg atgcgggtag tagatgtggg  1621 cagagacacc cacactgcca gatcttaata cgactcacta tagggagacc atgggtcctt  1681 acggacgctc cctgactgaa gggattaaga ccatttacag aacctgttcc acttaatgaa  1741 gttgcgtcct tacggacgct ccctgactga agggattaag acctcgaggc tgtggtctag  1801 acattccata catatcgggg gggtaggggt tttttgtgtg cctctagtgg ctggctaaga  1861 ataatacgac tcactatagg gagaggatcc ataaaggagg taaataatga actggcttgc  1921 cgcctcttgc catgtggctg cctttgcgct gttacataac ctgaaaccgc tggcgcagcg  1981 tgccggcatt caggatttcc cgtcaccggc gctggcaaac aaactgtttg cgccgctgcc  2041 ggaaagcctc tcggcccgtg cggatctgca cgaaaataca ttaaccctgc tattggattt  2101 cgccgccaga ctggcgcgcg gcctgccgga gacgcccgta agccgtggcg aaaccgggct  2161 gataccgctc acccacctgt tagcggacga taaccacagc gccccggcgg agcgctgtta  2221 cagcccactg gccccgctgg gggtcgattc gctgatgccg gtgacggcag cgttgcccac  2281 cggcgaacgg caggcggcgt accggcaggt gtatcaggca ttgatagagg ggctggaggc  2341 gatccccgcc gcccaccgcc tgcaaccgtc gctgtggctt gaccacctcg acagcctgtg  2401 gatgaccacc tgccacgcgc tgccagacgg cgacggggcc agcggtatct cgctgtacga  2461 tcagggcaaa accacggcgg cgctcgccgt cgcactgtgg cagcaccacg cccgtcaggc  2521 cacaccggag aaggaaattt cactcacgcc aggcgacgat gcgcgcctgc tgctgattca  2581 ggccgatgtg ttcggcattc aagagctgat tttcgcccag ggcaatcaga cgcaaaaaat  2641 ggccgccaaa ctgctgcgcg ggcgttcgtt tcaggtatcc ctgctggcgg aaaccgctgc  2701 gctgcgggtg ctggaaacct ttgacctgcc gccggtttgc cagttgatta acgccgccgg  2761 caaaagcctg attgtcgcgc cgaatctgcc ggatgccgcc gaacggctgg ccgggttgcg  2821 tcagcgtctt gatgagtggt ttttaaccca cacctacgcg caaaccggca tcgggctgtg  2881 cagcacggtg gccggcagcg ctgagttcct cggcgagcag gcttacggca ggctgcaaaa  2941 ccggctggcg caggcgatgg agcaacagaa ataccagcgt ttttccctgt gtgccggtga  3001 tgcgcccccg ccggtgtttg agggttatct ggaccaaata gcgcagggcg caggcggtga  3061 accctgcccg atgaatggcc tgcacccggt ggaaaccacc ctcaacgggc ttggctgtag  3121 ccgactggcg gcagaccaga taaccctcgg ccactggata aaccacggcc actggataag  3181 ccacggcgag cgttacgccc ggctgttgat cctgcgtgac agcaccgcca ccttctctga  3241 tgaccgctgc ctgcacctga cgctgtttgg ctatcaggtg gtggccgtca gcctgaaaga  3301 ggacagcggc gagttcggcg aactggcgtg caacggcagc ctgcgccgct gctgggatgt  3361 cagcctgccg ccggaaggca cctccctgtt tcagggttat gcccgccgtt ttatcaacgg  3421 ctggatgccg ctggcgcagg gggaatatca gccggagtgc taccccggca ttgaagaagc  3481 gctcgctaac ggcgacatca aaacctttga tcacctgagc tgtgaagatt tgtatcagga  3541 tgccgaccag tcgttacgcg gcacctgcgc gctgggcgtg ctgaaagggg atatcgacaa  3601 cctcggccac ctgtttcgca gcggcctgcc gcaaccgggc tttgccaaaa ccatcggcct  3661 gtcgcgccag atacacctgt ttttcactct gtggctgccg catctgtgcc gcaaagaccc  3721 gcgttttgcc aacacctaca ccgtgttcgc cggcggcgac gatttctttt tgattggccc  3781 gtggcgcagc cagcaaacgc tggcgctaac aatggcgcag gacttcgcgc gctacagcgg  3841 ccacaatccg gcgctgcatt tttcactcgg gctggtgcag gccaaacccg gctacccggt  3901 gcgggcgctg gcggctcagg ccgaagccgc tctgaagcag gccaagcaac accccggcaa  3961 gaacgccatc tgcctgtata acgaggtgat gggctggccg gaatatgacg cgttactggc  4021 gtgcagtgag gagctggcgc gctggcgcga gcatgacggc tacccgctct cctccggcct  4081 gctttaccgg ttgctggcgc tcagtgaaca gtcggcggag gagtcagaaa aaccgcaggc  4141 ggcactgtgg cgcagccggc tggcctattt tttacgtcgc aatctggtgg ataacgtgaa  4201 agtcccgacg aaagaaaacc cggcagcgtt ccgccatcag cttcatttgg aattgtttga  4261 gaaacttgag cagcacctga aacggcaccg ccagcgttac cgggtggcgc ttcaacgcca  4321 tctctaccac taccgcacgg tgccgcgcgg cgagtaacat atggctgcgt ggtcaaatgt  4381 gcgtacccta accccttccc cggtcaatcg gggcggatgg ggttttttgt gcgtacttca  4441 ttatgtatat taatacgact cactataggg agaagatcta taaaggaggt aaataatgca  4501 tcaccatcac catcacacga cgctcgacta ttttaaacgc caactgcaca agccggaggc  4561 gacatttttt gatgacgacg cccaaaaatt tgccgaacag ttggctcaat acggtaagaa  4621 gggcaaaccc actcagttgc gccgctttta cgaccagtta cagcaactgg agcagcgcat  4681 caatggcgat gaggaaaaac tggctctcta cctacccgag atccgcatga tcagcgccca  4741 tctggcgtat gccaaagggc gcgagcttat ctctgacgag ttttgccaaa ccatgcaaaa  4801 cctgattcgc agcatcaaca cctgccagca cctgaaaaat ggccggctgt tttttgaagc  4861 caccctcggt tttttacgcg ccatacgccg cgactgaacg cgtgctgcgt ggtcaaatgt  4921 gcgtagacca accccttgcg gcctcaatcg ggggggatgg ggttttttgt caggcaagtc  4981 tcagctggtt taatacgact cactataggg agagaattca taaaggaggt aaataatgca  5041 actgaacaat atccagacgt tacgcgccac tctggtgtgt gaaaccgggt tacacattgg  5101 cggcggcgac accgcgttgc agattggcgg catcgacagc gccgtggtgc gccacccgtt  5161 gacccagcaa ccttacattc ccggctccag cctgaaaggc aaactgcgca gcctgctcga  5221 atggcgcgct ggtgtggtcg gcgataccga gggcaaggtg ctcagtcatc aggtttacca  5281 gcaactgatc gacgataaaa aacaggcaca ggcactacag gcactgaaaa tcctgcaact  5341 gtttggcgtc agcggcggcg acaagctttc cgccgaacaa gcccaacaga tcggcccgac  5401 tcgcctgtcc ttctgggatt gcgaatttga cgaacactgg ctggcgcagc aaggcgggcg  5461 cgtccagacc gaagagaaag cggaaaactg tattgaccgc atcagtggcg tggcgctgca  5521 cccgcgcttt atcgagcgtg tgcccgccgg cagccgcttt gactttcgcc tcaccgtgcg  5581 ccagcttgat ggcgacagcc ccgacctgct cgacaccctg ttggcgggcc tgaaaatgct  5641 ggagctggac gggctaggcg gcagcatttc ccgtgggtat ggcaaagtgc gctttgaagc  5701 gctgaccctc gacgggaaag atctccagcc gcgctttgag cagttacagc cgtttaaaca  5761 caccacgcag ggagccggat gaaagcttac ctggagatca aggagattac tctaacccca  5821 tcggccgtct taggggtttt ttgtcctgtg ttagctggag ggtataatac gactcactat  5881 agggagaccc gggataaagg aggtaaataa tgccctcccc cgttgccccc gccgccgcat  5941 ggcaatggct acgcctgcgg ctgctccccg acagcgcctt tgccaccgcg ctgcgcggtg  6001 acacgctgtt tggccagtta tgctggtatt tgcgcgaatc cctcggtgag gcgggcctga  6061 atgcgctgct ggcaggctac cacgaccagc gaccgttcgc ggtgatcagc gacccgatgc  6121 tggccgacca cctgccgcgc ccgcacctgc ccgaacaccg gctgggcttc gccgccgccg  6181 atgcccgcgc ccgcaaacaa cgtaaacagc aatgctggct gccgcttgcc ttcgctcacc  6241 agccgctggc cgagtggggc gcacacttaa ccgccgcccc tgagcaccac cagcaccgca  6301 gcgacgtgca gatgcacaac agcatcaacc gccagacact taccaccggt ggcgatgacg  6361 cgttcgcccc gtttggcagc gagcagcact ggtttgatgt ggacaccgcc tgggatctct  6721 tggcagacag cgccgccgtg ctggcccccg ttaccccgga taaccgcccg tttgtcggcc  6781 agggattagg cggcaatggc cgtctttcca ccgccctgcc acaaacggtg catcagggct  6841 atgcgccggt gatcccggtg cgctttcacc ataaggcaca gccacaatga tcatgatttc  6901 ttgtcgaact ggacagtagc agaaccgcta acgggggcga aggggttttt tgtgacatac  6961 gagctgattg aactaatacg actcactata gggagaggta ccataaagga ggtaaataat  7021 gacccgcgac cagacgccac gtcgccacac cgcctgtgac agcgacaaca tgcaatactt  7081 taccctcacc tgcctgtcgc cggtgcatgt cgccaccggc gacagcctga accccggtga  7141 atacctgata gacgaaaatg cactgtatga actggggcaa ggtggtctga gcccggcgct  7201 cacggcgaca cagcgcagcg agttgctcac tattctggag agcaatgacc ctgctctgcc  7261 gctgaccgtg cagcgttttc tggcacggga agcgggcaag ctgaaatacg ccgcccgccg  7321 gatgtgcccg ctgttacccg gcatcagccg ttattaccag tcacggctcg ggcaggtgat  7381 gcagaacgat accaaaaaca aaaagcagat gatcaaccag ctagagctga tgcgccacgt  7441 cggggccgca ctgggcgcgc cttatattcc cggatctacg ctcaaagggg ctatccgcac  7501 cgcactggtc agcgcgctca atcaaggcca gccactgcaa gccgagcgac gggaaaccga  7561 caaactgagc agcaaggtgg cgcaggatgc ggaacgccag ctactgggct ttgatacccg  7621 ccgcgacagc ccgcgatacc gcattgagca tgaccccttc cactggctac aggtggggga  7681 tgccgtaagc ccggcagagc acccgcccat gctggattac tggctggtac gccgccagcc  7741 gttcaaacgc accgagaagc aggacaacaa ggcggacaat atggaactgt cgccggttga  7801 atgccttaaa ccccggcaaa gcccgttgca ctgccagata accgtgaaaa cgccgcccac  7861 cgctctcgca ataaaaaacc cgcgcctgaa gcagtggctt ggcaaggtgt cgcaactggc  7921 gcaacaggtg aacaggataa ccctgccgca atgccatcac gagctggcat ggctggccga  7981 aaagcacatc ggcacggatg atgtgtacgc ccccggccag aactgggtgg cgcagatgca  8041 gcaattactg cgccagttag acgacccgct acagcgcggc gaggcactct tgctgcgggt  8101 gggcaaatac ggcggagcca tcagcaaaac cgtggcaggc tggcggcata tcgcccggct  8161 ggggagacaa ggcacgcgca ccacctacca cccggacgtc accacctgca cgctggcgct  8221 gccgcaagct gatgcgctga cacaggcgct gccctttggc tgggtactgt tgcaccagcc  8281 tgaccagccg gaggtgaccg agtttgtcgc cagccatcat gactggtgcc agcaacagca  8341 acagcggctt gacgcgcatc agcaacagca gcacacccac cgccagcaac gccagcagtt  8401 ggctcaggct cgtgaagagg aagcgcagcg gctggcagac aaagcccgcc agagcaaagc  8461 gcgccagtcc atcatgtcgc tggcagaaca actggcgagt gagcaaacgt ttcagcataa  8521 aaaccccaac ggcccgctgc gcggtcagtt agccacctgt gtgggttgcg ttgccactga  8581 aggctcagca gaagagaaag ccgagttgtg cacactgttt gacgacatcc tcaactattg  8641 gggcatcaaa cccggcaaag ataaaaagct cacggccttg aggaataagc tgttatgacc  8701 taggcgcttc aacggaacgg atcttacata tcgggggggt aggggttttt tgtctcggag  8761 accaagtagg gcataatacg actcactata gggagactat ggataaagga ggtaaataat  8821 gactaatcag gcaaaaaagt tatctagaat taatggtagg gagtttttaa aacagtcctt  8881 taatttacaa caacaactat tggcctctca attaaattta tcccgaacga ttacgcatga  8941 tggaacgatg ggggaggtta atgaaagtta ttttttgagt attatccgcc agtatttgcc  9001 tgaacgttac tcggttgacc ggggagttgt ggtggattca gaaggccaga ccagcgacca  9061 gatagatgca gtgatttttg accggcatta cacaccgaca ttattagacc aacaagggca  9121 caggtttatt ccggcagagg cggtgtacgc ggtactggag gttaaaccaa ccattaataa  9181 aacctacctt gaatatgcag ccgataaagc tgcatctgtc cgaaaattat atcgaaccag  9241 tacggtaata aaaaatattt acggtacggc caaaccggtc gaacatttcc cgatcgtagc  9301 aggtattgtg gcgattgatg ttgagtggca agacggactc ggaaaggcat ttactgaaaa  9361 tttgcaggct gtttccagcg atgaaaaccg aaaactggat tgcggtctgg cggtgtcggg  9421 cgcatgtttt gatagttatg atgaggaaat aaaaatcaga agcggtgaaa atgcattaat  9481 cttttttctg ttccgtttgc tcggtaaatt gcaatcatta ggtacggtgc ccgcaattga  9541 ctggcgggtg tatatagata gtctggaata actcgagagg ttacagcctg cataatgtag  9601 cataacccct tggggcctct aaacgggtct tgaggggttt tttgtgccta tagtttgaag  9661 cagaatcgaa tttctgccat tcatccgctt attatcactt attcaggcgt agcaccaggc  9721 gtttaagggc accaataact gccttacaaa aaacccctag ccgcccgata agagcgggct  9781 aggggttcga gtaaaaaaaa ttacgccccg ccctgccact catcgcagta ctgttgtaat  9841 tcattaagca ttctgccgac atggaagcca tcacagacgg catgatgaac ctgaatcgcc  9901 agcggcatca gcaccttgtc gccttgcgta taatatttgc ccatcgtgaa aacgggggcg  9961 aagaagttgt ccatattggc cacgtttaaa tcaaaactgg tgaaactcac ccagggattg  10021 gctgaaacga aaaacatatt ctcaataaac cctttaggga aataggccag gttttcaccg  10081 taacacgcca catcttgcga atatatgtgt agaaactgcc ggaaatcgtc gtggtattca  10141 ctccagagcg atgaaaacgt ttcagtttgc tcatggaaaa cggtgtaaca agggtgaaca  10201 ctatcccata tcaccagctc accgtctttc attgccatac ggaactccgg gtgagcattc  10261 atcaggcggg caagaatgtg aataaaggcc ggataaaact tgtgcttatt tttctttacg  10321 gtctttaaaa aggccgtaat atccagctga acggtctggt tataggtaca ttgagcaact  10381 gactgaaatg cctcaaaatg ttctttacga tgccattggg atatatcaac ggtggtatat  10441 ccagtgattt ttttctccat tttagcttcc ttagctcctg aaaatctcga taactcaaaa  10501 aatacgcccg gtagtgatct tatttcatta tggtgaaagt tggaacctct tacgtgccga  10561 tcaaggtctc attttcgcca aaagttggcc cagggcttcc cggtatcaac agggacacca  10621 ggatttattt attctgcgaa gtgatcttcc gtcacaggta tttattcggc gcaaagtgcg  10681 tcgggtgatg ctgccaactt actgatttag tgtatgatgg tgtttttgag gtgctccagt  10741 ggcttctgtt tctatcagct gtccctcctg ttcagctact gacggggtgg tgcgtaacgg  10801 caaaagcacc gccggacatc agcgctagcg gagtgtatac tggcttacta tgttggcact  10861 gatgagggtg tcagtgaagt gcttcatgtg gcaggagaaa aaaggctgca ccggtgcgtc  10921 agcagaatat gtgatacagg atatattccg cttcctcgct cactgactcg ctacgctcgg  10981 tcgttcgact gcggcgagcg gaaatggctt acgaacgggg cggagatttc ctggaagatg  11041 ccaggaagat acttaacagg gaagtgagag ggccgcggca aagccgtttt tccataggct  11101 ccgcccccct gacaagcatc acgaaatctg acgctcaaat cagtggtggc gaaacccgac  11161 aggactataa agataccagg cgtttccccc tggcggctcc ctcgtgcgct ctcctgttcc  11221 tgcctttcgg tttaccggtg tcattccgct gttatggccg cgtttgtctc attccacgcc  11281 tgacactcag ttccgggtag gcagttcgct ccaagctgga ctgt | |
| pTarget | 1 gtttgacagc ttatcatcga ctgcacggtg caccaatgct tctggcgtca ggcagccatc  61 ggaagctgtg gtatggctgt gcaggtcgta aatcactgca taattcgtgt cgctcaaggc  121 gcactcccgt tctggataat gttttttgcg ccgacatcat aacggttctg gcaaatattc  181 tgaaatgagc tgttgacaat taatcatccg gctcgtataa tgtgtggaat tgtgagcgga  241 taacaatttc acacaggaaa cagcgccgct gagaaaaagc gaagcggcac tgctctttaa  301 caatttatca gacaatctgt gtgggcactc gaccggaatt atcgattaac tttattatta  361 aaaattaaag aggtatatat taatgtatcg attaaataag gaggaataaa ccatgggggg  421 ttctcatcat catcatcatc atggtatggc tagcatgact ggtggacagc aaatgggtcg  481 ggatctgtac gacgatgacg ataaggatcc aacccttttc caagcttgca acttcattaa  541 gtggaacagg ttctgtaaat gcaagagcag catgcttcca aggcgaattc gaagcttggc  601 tgttttggcg gatgagagaa gattttcagc ctgatacaga ttaaatcaga acgcagaagc  661 ggtctgataa aacagaattt gcctggcggc agtagcgcgg tggtcccacc tgaccccatg  721 ccgaactcag aagtgaaacg ccgtagcgcc gatggtagtg tggggtctcc ccatgcgaga  781 gtagggaact gccaggcatc aaataaaacg aaaggctcag tcgaaagact gggcctttcg  841 ttttatctgt tgtttgtcgg tgaacgctct cctgagtagg acaaatccgc cgggagcgga  901 tttgaacgtt gcgaagcaac ggcccggagg gtggcgggca ggacgcccgc cataaactgc  961 caggcatcaa attaagcaga aggccatcct gacggatggc ctttttgcgt ttctacaaac  1021 tcttttgttt atttttctaa atacattcaa atatgtatcc gctcatgaga caataaccct  1081 gataaatgct tcaataatat tgaaaaagga agagtatgag tattcaacat ttccgtgtcg  1141 cccttattcc cttttttgcg gcattttgcc ttcctgtttt tgctcaccca gaaacgctgg  1201 tgaaagtaaa agatgctgaa gatcagttgg gtgcacgagt gggttacatc gaactggatc  1261 tcaacagcgg taagatcctt gagagttttc gccccgaaga acgttttcca atgatgagca  1321 cttttaaagt tctgctatgt ggcgcggtat tatcccgtgt tgacgccggg caagagcaac  1381 tcggtcgccg catacactat tctcagaatg acttggttga gtactcacca gtcacagaaa  1441 agcatcttac ggatggcatg acagtaagag aattatgcag tgctgccata accatgagtg  1501 ataacactgc ggccaactta cttctgacaa cgatcggagg accgaaggag ctaaccgctt  1561 ttttgcacaa catgggggat catgtaactc gccttgatcg ttgggaaccg gagctgaatg  1621 aagccatacc aaacgacgag cgtgacacca cgatgcctgt agcaatggca acaacgttgc  1681 gcaaactatt aactggcgaa ctacttactc tagcttcccg gcaacaatta atagactgga  1741 tggaggcgga taaagttgca ggaccacttc tgcgctcggc ccttccggct ggctggttta  1801 ttgctgataa atctggagcc ggtgagcgtg ggtctcgcgg tatcattgca gcactggggc  1861 cagatggtaa gccctcccgt atcgtagtta tctacacgac ggggagtcag gcaactatgg  1921 atgaacgaaa tagacagatc gctgagatag gtgcctcact gattaagcat tggtaactgt  1981 cagaccaagt ttactcatat atactttaga ttgatttaaa acttcatttt taatttaaaa  2041 ggatctaggt gaagatcctt tttgataatc tcatgaccaa aatcccttaa cgtgagtttt  2101 cgttccactg agcgtcagac cccgtagaaa agatcaaagg atcttcttga gatccttttt  2161 ttctgcgcgt aatctgctgc ttgcaaacaa aaaaaccacc gctaccagcg gtggtttgtt  2221 tgccggatca agagctacca actctttttc cgaaggtaac tggcttcagc agagcgcaga  2281 taccaaatac tgtccttcta gtgtagccgt agttaggcca ccacttcaag aactctgtag  2341 caccgcctac atacctcgct ctgctaatcc tgttaccagt ggctgctgcc agtggcgata  2401 agtcgtgtct taccgggttg gactcaagac gatagttacc ggataaggcg cagcggtcgg  2461 gctgaacggg gggttcgtgc acacagccca gcttggagcg aacgacctac accgaactga  2521 gatacctaca gcgtgagcta tgagaaagcg ccacgcttcc cgaagggaga aaggcggaca  2581 ggtatccggt aagcggcagg gtcggaacag gagagcgcac gagggagctt ccagggggaa  2641 acgcctggta tctttatagt cctgtcgggt ttcgccacct ctgacttgag cgtcgatttt  2701 tgtgatgctc gtcagggggg cggagcctat ggaaaaacgc cagcaacgcg gcctttttac  2761 ggttcctggc cttttgctgg ccttttgctc acatgttctt tcctgcgtta tcccctgatt  2821 ctgtggataa ccgtattacc gcctttgagt gagctgatac cgctcgccgc agccgaacga  2881 ccgagcgcag cgagtcagtg agcgaggaag cggaagagcg cctgatgcgg tattttctcc  2941 ttacgcatct gtgcggtatt tcacaccgca tatggtgcac tctcagtaca atctgctctg  3001 atgccgcata gttaagccag tatacactcc gctatcgcta cgtgactggg tcatggctgc  3061 gccccgacac ccgccaacac ccgctgacgc gccctgacgg gcttgtctgc tcccggcatc  3121 cgcttacaga caagctgtga ccgtctccgg gagctgcatg tgtcagaggt tttcaccgtc  3181 atcaccgaaa cgcgcgaggc agcagatcaa ttcgcgcgcg aaggcgaagc ggcatgcatt  3241 tacgttgaca ccatcgaatg gtgcaaaacc tttcgcggta tggcatgata gcgcccggaa  3301 gagagtcaat tcagggtggt gaatgtgaaa ccagtaacgt tatacgatgt cgcagagtat  3361 gccggtgtct cttatcagac cgtttcccgc gtggtgaacc aggccagcca cgtttctgcg  3421 aaaacgcggg aaaaagtgga agcggcgatg gcggagctga attacattcc caaccgcgtg  3481 gcacaacaac tggcgggcaa acagtcgttg ctgattggcg ttgccacctc cagtctggcc  3541 ctgcacgcgc cgtcgcaaat tgtcgcggcg attaaatctc gcgccgatca actgggtgcc  3601 agcgtggtgg tgtcgatggt agaacgaagc ggcgtcgaag cctgtaaagc ggcggtgcac  3661 aatcttctcg cgcaacgcgt cagtgggctg atcattaact atccgctgga tgaccaggat  3721 gccattgctg tggaagctgc ctgcactaat gttccggcgt tatttcttga tgtctctgac  3781 cagacaccca tcaacagtat tattttctcc catgaagacg gtacgcgact gggcgtggag  3841 catctggtcg cattgggtca ccagcaaatc gcgctgttag cgggcccatt aagttctgtc  3901 tcggcgcgtc tgcgtctggc tggctggcat aaatatctca ctcgcaatca aattcagccg  3961 atagcggaac gggaaggcga ctggagtgcc atgtccggtt ttcaacaaac catgcaaatg  4021 ctgaatgagg gcatcgttcc cactgcgatg ctggttgcca acgatcagat ggcgctgggc  4081 gcaatgcgcg ccattaccga gtccgggctg cgcgttggtg cggatatctc ggtagtggga  4141 tacgacgata ccgaagacag ctcatgttat atcccgccgt taaccaccat caaacaggat  4201 tttcgcctgc tggggcaaac cagcgtggac cgcttgctgc aactctctca gggccaggcg  4261 gtgaagggca atcagctgtt gcccgtctca ctggtgaaaa gaaaaaccac cctggcgccc  4321 aatacgcaaa ccgcctctcc ccgcgcgttg gccgattcat taatgcagct ggcacgacag  4381 gtttcccgac tggaaagcgg gcagtgagcg caacgcaatt aatgtaagtt agcgcgaatt  4441 gatctg | |
| Name | Sequence (5’-3’) | Description |
| Gene fragments | |  |
| dPalm fragment | GCCAAAACCATCGGCCTGTCGCGCCAGATACACCTGTTTTTCA  CTCTGTGGCTGCCGCATCTGTGCCGCAAAGACCCGCGTTTTGC  CAACACCTACACCGTGTTCGCCGCAGCGGCCGCATTCTTTTTG  ATTGGCCCGTGGCGCAGCCAGCAAACGCTGGCGCTAACAATGG  CGCAGGACTTCGCGCGCTACAGCGGCCACAATCCGGCGCTGCA  TTTTTCACTCGGGCTGGTGCAGGCCAAACCCGGCTACCCGGTG  CGGGCGCTGGCGGCTCAGGCCGAAGCCGCTCTGAAGCAGGCCA  AGCAACACCCCGGCAAGAACGCCATCTGCCTGTATAACGAGGT  GATGGGCTGGCCGGAATATGACGCGTTACTGGCGTGCAGTGAG  GAGCTGGCGCGCTGGCGCGAGCATGACGGCTACCCGCTCTCCT  CCGGCCTGCTTTACCGGTTGCTGGCGCTCAGTGAACAGTCGGC  GGAGGAGTCAGAAAAACCGCAGGCGGCACTGTGGCGCAGCCGG  CTGGCCTATTTTTTACGTCGCAATCTGGTGGATAACGTGAAAG  TCCCGACGAAAGAAAACCCGGCAGCGTTCCGCCATCAGCTTCA  TTTGGAATTGTTTGAGAAACTTGAGCAGCACCTGAAACGGCAC  CGCCAGCGTTACCGGGTGGCGCTTCAACGCCATCTCTACCACT  ACCGCACGGTGCCGCGCGGCGAGTAACATAT | Mutates GGDD motif of Cas10 Palm2 domain to AAAA |
| dCsm3 fragment | ATGCAACTGAACAATATCCAGACGTTACGCGCCACTCTGGTG  TGTGAAACCGGGTTACACATTGGCGGCGGCGACACCGCGTTG  CAGATTGGCGGCATCGCCAGCGCCGTGGTGCGCCACCCGTTG  ACCCAGCAACCTTACATTCCCGGCTCCAGCCTGAAAGGCAAA  CTGCGCAGCCTGCTCGAATGGCGCGCTGGTGTGGTCGGCGAT  ACCGAGGGCAAGGTGCTCAGTCATCAGGTTTACCAGCAACTG  ATCGACGATAAAAAACAGGCACAGGCACTACAGGCACTGAAA  ATCCTGCAACTGTTTGGCGTCAGCGGCGGCGACAAGCTTTCC  GCCGAACAAGCCCAACAGATCGGCCCGACTCGCCTGTCCTTC  TGGGATTGCGAATTTGACGAACACTGGCTGGCGCAGCAAGGC  GGGCGCGTCCAGACCGAAGAGAAAGCGGAAAACTGTATTGAC  CGCATCAGTGGCGTGGCGCTGCACCCGCGCTTTATCGAGCGT  GTGCCCGCCGGCAGCCGCTTTGACTTTCGCCTCACCGTGCGC  CAGCTTGATGGCGACAGCCCCGACCTGCTCGACACCCTGTTG  GCGGGCCTGAAAATGCTGGAGCTGGACGGGCTAGGCGGCAGC  ATTTCCCGTGGGTATGGCAAAGTGCGCTTTGAAGCGCTGACC  CTCGACGGGAAAGATCTCCAGCCGCGCTTTGAGCAGTTACAG  CCGTTTAAACACACCACGCAGGGAGCCGGATGA | Introduces D34A mutant to Csm3 |
| B01 fragment | TAATACGACTCACTATAGGGAGACCATGGGTCCTTACGGACG  CTCCCTGACTGAAGGGATTAAGACTCCGGAGTCAGATGCACG  ATGGTGTCTGTTTGAGGTCCTTACGGACGCTCCCTGACTGAAGG  GATTAAGACCTCGAGGCTGTGGTCTAGACA | Cloning spacer B01, for expression of crRNA-B01 |
| S01 fragment | 5’TAATACGACTCACTATAGGGAGACCATGGGTCCTTACGGACGCT  CCCTGACTGAAGGGATTAAGACACAGGAGTCAGATGCACGATGGT  GTCTGTTTGAGGTCCTTACGGACGCTCCCTGACTGAAGGGATTAAG  ACCTCGAGGCTGTGGTCTAGACA | Cloning spacer S01, for expression of crRNA-S01 |
| Oligonucleotides | |  |
| prCCP_077 | AATTCTGTTTTATCAGACCGTTATTCCAGACTATCTATATACACCCGCC | Rev primer to add pBAD homology to NucC |
| prCCP_078 | GGCTAACAGGAGGAATTAACATGACTAATCAGGCAAAAAAGTTATCTAGAAT | Fwd primer to add pBAD homology to NucC |
| prCCP_079 | GTTAATTCCTCCTGTTAGCCCAAAAAACGG | Rev primer to amplify region of interest from pBAD |
| prCCP_080 | CGGTCTGATAAAACAGAATTTGCCTGGC | Fwd primer to amplify region of interest from pBAD |
| prCCP_085 | GCAGCAGTGATTTTTGACCGGCATTACACA | Rev primer to introduce D83Q mutation in NucC |
| prCCP_086 | TATCTGGTCGCTGGTCTGGC | Fwd primer to introduce D83Q mutation in NucC |
| prCCP_087 | GCAGTTAAACCAACCATTAATAAAACCTACCTTGAATATG | Rev primer to introduce E114A mutation in NucC |
| prCCP_088 | CAGTACCGCGTACACCGCCTC | Fwd primer to introduce E114A mutation in NucC |
| prCCP_101 | CCACGCAGGGAGCCGGATGA | Rev primer to copy pACYC-Csm region of interest for Gibson assembly |
| prCCP_102 | TGGATATTGTTCAGTTGCATTATTTACCTCCTTTATGA | Fwd primer to copy pACYC-Csm region of interest for Gibson assembly |
| prCCP_103 | GTGCCGCGCGGCGAGTAACATATG | Rev primer to copy pACYC-Csm region of interest for Gibson assembly |
| prCCP_104 | CGACAGGCCGATGGTTTTGGC | Fwd primer to copy pACYC-Csm region of interest for Gibson assembly |
| prCCP_109 | AGCGACGCAATAGATGCAGT | Fwd primer to introduce Q81A into pBAD NucC |
| prCCP_110 | GGTCTGGCCTTCTGAATCCA | Rev primer to introduce Q81A into pBAD NucC |
| prCCP_121 | CGTTCCGTTGAAGCGCCTAGGTC | Rev primer to make pACYC-Csm ΔΝucC |
| prCCP_122 | GAATAACTCGAGAGGTTACAGCCTGCATAAT | Fwd primer to make pACYC-Csm ΔΝucC |
| prCCP_185 | CACCGCGTTGGCGATTGGCGGCATCG | Fwd primer to make Q29A Csm3 |
| prCCP_186 | TCGCCGCCGCCAATGTGT | Rev primer to make Q29A Csm3 |
| prCCP_187 | CGACACCGCGGCGCAGATTGGCG | Fwd primer to make L28A Csm3 |
| prCCP_188 | CCGCCGCCAATGTGTAAC | Rev primer to make L28A Csm3 |
| prCCP_189 | GCGGGCGGCATCGACAGCG | Fwd primer to make I30A Csm3 |
| prCCP_190 | CTGCAACGCGGTGTCG | Rev primer to make I30A Csm3 |
| prCCP_191 | TGCGCTGTTAGCGAACCTGAAACCGC | Fwd primer to make H17A Cas10 |
| prCCP_192 | AAGGCAGCCACATGG | Rev primer to make H17A Cas10 |
| prIVT_001 | GAATTCTAATACGACTCACTATAGGGCAACTTCATTAAGTGGAA | Primer for overlap extension PCR to generate mismatch targets |
| prIVT_002 | TACTTCCGCATTTACAGAACCTGTTCCACTTAATGAAGTTGC | Pairs with prIVT_001 to generate cognate target |
| prIVT_003 | TACTTCCGGATTTACAGAACCTGTTCCACTTAATGAAGTTGC | Pairs with prIVT_001 to generate +1 mismatch |
| prIVT_004 | TACTTCCGCTTTTACAGAACCTGTTCCACTTAATGAAGTTGC | Pairs with prIVT_001 to generate +2 mismatch |
| prIVT_005 | TACTTCCGCAATTACAGAACCTGTTCCACTTAATGAAGTTGC | Pairs with prIVT_001 to generate +3 mismatch |
| prIVT_006 | TACTTCCGCATATACAGAACCTGTTCCACTTAATGAAGTTGC | Pairs with prIVT_001 to generate +4 mismatch |
| prIVT_007 | TACTTCCGCATTAACAGAACCTGTTCCACTTAATGAAGTTGC | Pairs with prIVT_001 to generate +5 mismatch |
| prIVT_008 | TACTTCCGCATTTTCAGAACCTGTTCCACTTAATGAAGTTGC | Pairs with prIVT_001 to generate +6 mismatch |
| prIVT_009 | TACTTCCGCATTTAGAGAACCTGTTCCACTTAATGAAGTTGC | Pairs with prIVT_001 to generate +7 mismatch |
| prIVT_010 | TACTTCCGCATTTACTGAACCTGTTCCACTTAATGAAGTTGC | Pairs with prIVT_001 to generate +8 mismatch |
| prIVT_011 | TACTTCCGCATTTACACAACCTGTTCCACTTAATGAAGTTGC | Pairs with prIVT_001 to generate +9 mismatch |
| prIVT_012 | TACTTCCGCATTTACAGTACCTGTTCCACTTAATGAAGTTGC | Pairs with prIVT_001 to generate +10 mismatch |
| prIVT_013 | TACTTCCGCATTTACAGATCCTGTTCCACTTAATGAAGTTGC | Pairs with prIVT_001 to generate +11 mismatch |
| ssRNA-d02  Cognate | GCAACUUCAUUAAGUGGAACAGGUUCUGUAAAUGCGGAAGUA |  |
| ssRNA-d03  Non-cognate | GCAACUUCAUUAAGUGGAACAGGUUCUGUAAAUGGUCUUAAU |  |
| HBB target | CUCAAACAGACACCAUGGUGCAUCUGACUCCUGAGGAGAAGUCUGCCG | Synthetic RNA mimicking *HBB* transcript |
| A20T target | CUCAAACAGACACCAUGGUGCAUCUGACUCCUGUGGAGAAGUCUGCCG | Synthetic RNA mimicking the A20T transcript |
| Reporter DNA 1 | /56-FAM/TCAAAGGCGCCCTTGGTACATACACGATTT | Cleaved by NucC in fluorogenic assay |
| Reporter DNA 2 | AAATCGTGTATGTACCAAGGGCGCCTTTGA/IBFQ/ | Cleaved by NucC in fluorogenic assay |

**Table S2. Buffers used in Cas10-Csm purification**

| Name | Reagent |
| --- | --- |
| Phosphate lysis buffer | 100 mM NaH_2_PO_4_ pH 8.0 |
|  | 600 mM NaCl |
|  | 20 mM imidazole |
|  | 1 mg/mL lysozyme |
|  | 2 mM PMSF |
|  | 0.1% (v/v) triton X-100 |
|  | 1% v/v glycerol |
|  | 1 mM DTT |
|  | 500 μM EDTA |
| Phosphate wash buffer 1 | 100 mM NaH_2_PO_4_ |
|  | 150 mM NaCl |
|  | 1 mM DTT |
|  | 1% (v/v) glycerol |
|  | 20 mM imidazole |
| Phosphate wash buffer 2 | Phosphate wash buffer 1 with 40 mM imidazole |
| Phosphate elution buffer 1 | Phosphate wash buffer 1 with 100 mM imidazole |
| Phosphate elution buffer 2 | Phosphate wash buffer 1 with 250 mM imidazole |
| Tris lysis buffer | 50 mM Tris-HCl pH 8.0 |
|  | 300 mM NaCl |
|  | 20 mM imidazole |
|  | 1 mg/ml lysozyme |
|  | 1 mM PMSF |
|  | 0.1% (v/v) triton X-100 |
| Tris wash buffer | Tris lysis buffer with 40 mM imidazole and 10% (v/v) glycerol |
| Tris elution buffer 1 | Tris lysis buffer with 100 mM imidazole and 10% (v/v) glycerol |
| Tris elution buffer 2 | Tris lysis buffer with 250 mM imidazole and 10% (v/v) glycerol |
| Storage buffer | 50 mM Tris HCl pH 8.0 |
|  | 20 mM NaCl |
|  | 5% (v/v) glycerol |
| Reaction buffer 1 | 50 mM Tris HCl pH 8.0 |
|  | 150 mM NH_4_Cl |
|  | 5% (v/v) glycerol |
|  | 10 mM MgCl_2_ |
| Reaction buffer 2 | 50 mM Tris HCl pH 8.0 |
|  | 150 mM NaCl |
|  | 5% (v/v) glycerol |
|  | 10 mM MgCl_2_ |
| Reaction buffer 3 | 12.5 mM Tris HCl pH 8.0 |
|  | 20 mM NaCl |
|  | 10 mM MgCl_2_ |
|  | 10% v/v glycerol |
| EM buffer | 50 mM Tris HCl pH 8.0 |
|  | 100 mM NaCl |
|  | 1 mM MgSO_4_ |
|  | 4 mM CaCl_2_ |

**Table S3. Mass spectrometry identification of proteins in SerrCas10-Csm ultracentrifugation fractions.**

| Protein | Theoretical mass (kDa) | UNIPARC Identifiers | Unique peptide counts | PSMs^a^ | Sum PEP score |
| --- | --- | --- | --- | --- | --- |
| Cas10 | 90.2 | UPI0003926B6B | 29 | 358 | 80.165 |
| Csm5 | 63 | UPI0003921A6A | 21 | 431 | 80.144 |
| ArnA | 74.2 | UPI0000000F69 | 26 | 96 | 64.091 |
| Csm3 | 27.2 | UPI00039276A8 | 19 | 892 | 53.839 |
| Csm2 | 15.8 | UPI000392639F | 13 | 583 | 39.57 |
| Csm4 | 35.7 | UPI001F2BC94D | 10 | 219 | 39.294 |
| GroEL | 57.3 | UPI0000000ED4 | 14 | 32 | 30.367 |
| AdhE | 96.1 | UPI0000000054 | 16 | 37 | 29.725 |
| SlyD | 20.8 | UPI0000135A43 | 5 | 19 | 25.797 |

^a^ All proteins comprising 1% or more of total protein as determined by number PSM are reported.

PSM - Peptide spectrum match

PEP - Posterior error probability for peptide spectrum matches, PEP score is the negative logarithm of the PEP value

**Figure S3. MALDI mass spectrometry of cOA synthesis reactions plus and minus ATP.** Four major peaks are labeled: 986.238 corresponds to cA_3_ and 1315.296 corresponds to cA_4_. The non-specific peaks are those that appear in both the - ATP and +ATP reactions and therefore are not products of an ATP dependent reaction.

**Table S4. Cryo-EM Statistics for Data Collection and Model Quality**

| Data collection and processing | **Cas10-Csm unbound to target RNA**  **9ZS2**  **EMD-74675** | **Cas10-Csm bound to target RNA**  **9ZS4**  **EMD-74688** |
| --- | --- | --- |
| Magnification | 150,000 | 150,000 |
| Voltage (kV) | 200 | 200 |
| Electron exposure (e^-^/ Å^2^) | 40.0 | 40.0 |
| Defocus range (μm) | 0.1-3.5 | 0.1-3.0 |
| Pixel size (Å) | 0.94 | 0.94 |
| Initial particles (no.) | 593,739 | 324,865 |
| Final particles (no.) | 42,471 | 129,762 |
| Map resolution (Å) | 4.4 | 3.8 |
| FSC threshold | 0.143 | 0.143 |
| Map sharpening B factor (Å^2^) | 118.6 | 110.9 |
| Refinement |  |  |
| Model resolution (Å) | 4.4 | 3.8 |
| FSC threshold | 0.143 | 0.143 |
| Model composition |  |  |
| Nonhydrogen atoms | 18,543 | 23,918 |
| Protein residues | 2308 | 2867 |
| RNA residues | 30 | 64 |
| Bonds (RMSD) |  |  |
| Bond lengths (Å) | 0.003 | 0.003 |
| Bond angles (°) | 0.770 | 0.742 |
| Validation |  |  |
| Molprobity score | 1.99 | 2.00 |
| Clashscore | 15.68 | 9.85 |
| Ramachandran plot (%) |  |  |
| Outliers | 0.22 | 0.00 |
| Allowed | 4.02 | 3.34 |
| Favored | 95.76 | 96.66 |
| B factors, mean (Å^2^) |  |  |
| Protein | 420.62 | 203.32 |
| RNA | 313.83 | 148.32 |

**Figure S4. Workflow for single particle reconstructions of SerrCas10-Csm by cryo-EM.** Both structures (unbound to target, target RNA bound) were determined using a subset of the highest quality exposures. Iterative particle picking and 2D classification were performed in both cases using CryoSPARC version 4 followed by ab-initio volume reconstruction and non-uniform (NU) volume reconstruction. Gold Standard Fourier Shell Correlation (GSFSC) was 4.48 Å for the target unbound volume and 3.84 Å for the target RNA bound volume.

**Figure S5. Density for the crRNA-target duplex indicates the target is uncleaved.** Density for the crRNA-target RNA duplex at contour level 0.13. Target cleavage occurs at the flipped nucleotides (+6, +12, +18, +24).

**Figure S6. Local resolution data for SerrCas10-Csm bound to target.** (A) Local resolution plot for the target RNA bound complex. (B) Local resolution plots for individual components.

**Figure S7. Density quality for the SerrCas10-Csm complex bound to target.** Density is shown for each component on the right and the model fit to density is shown on the left.

**Figure S8. Local resolution data for SerrCas10-Csm unbound to target.** (A) Local resolution plot for the complex without target RNA. (B) Local resolution plots for individual components.

**Figure S9. Density quality for the SerrCas10-Csm complex unbound to target.** Density is shown for each component on the right and the model fit to density is shown on the left.

**Figure S10. Van der Waals networks in target RNA bound Cas10-Csm complexes from *Serratia sp.* ATCC 39006*, S. thermophilus* and *L. lactis*.** (A) A network of van der Waals interactions between Palm2, domain 4 and Csm3 residues is shown from the structure of *Serratia sp.* ATCC 39006 Cas10-Csm bound to target RNA. (B) The same region from *S. thermophilus* or (C) *L. lactis* Cas10-Csm bound to target RNA is shown. Both *S. thermophilus* and *L. lactis* complexes engage in Van der Waals interactions between Csm3, domain 4 and Palm2 in a manner similar, but not identical to *Serratia*.

**Figure S11. Performance of crRNA-B00 on HBB and A20T transcripts.** (Upper) A diagram of crRNA-B00 which pairs perfectly to an HBB transcript but forms a U-U mispair to the A20T transcript. (Lower) Despite the U-U mispair, Cas10-Csm complexes with crRNA-B00 still produce cOA stimulating the downstream DNA cleavage reaction and fluorescence at unacceptable levels. The reaction was performed with 200 nM HBB or A20T target RNA. All other reaction conditions are as reported in the Methods.

**Table S5. Fluorescence signal for titrations of wild type *HBB* RNA and variant**

| crRNA-B01/wt *HBB* RNA |  | | | |  |
| --- | --- | --- | --- | --- | --- |
| Concentration | **Raw fluorescence** |  | **Background subtracted intensity** | **I/σ(I)** | |
|  | **Mean** | **σ** | **Mean** |  | |
| 1 uM | 11459 | 541 | 8926 | 16.5 | |
| 100 nM | 10698 | 783 | 8166 | 10.4 | |
| 10 nM | 11084 | 1163 | 8551 | 7.4 | |
| 1 nM | 4036 | 267 | 1504 | 5.6 | |
| 100 pM | 2624 | 203 | 92 | 0.5 | |
| 10 pM | 2520 | 147 | -12 | -0.1 | |
| 1 M | 2382 | 215 | -151 | -0.7 | |
|  | Blank mean 2533 Blank s = 197 | | | |  |
|  |  |  |  |  | |
| crRNA-B01/A20T RNA |  | | | |  |
| Concentration | **Raw fluorescence** |  | **Background subtracted intensity** | **I/σ(I)** | |
|  | **Mean** | **σ** | **Mean** |  | |
| 1 uM | 10496 | 769 | 7986 | 10.4 | |
| 100 nM | 4219 | 492 | 1709 | 3.5 | |
| 10 nM | 2708 | 152 | 198 | 1.3 | |
| 1 nM | 2397 | 168 | -113 | -0.7 | |
| 100 pM | 2624 | 120 | 115 | 1.0 | |
| 10 pM | 2066 | 105 | -444 | -4.2 | |
| 1 pM | 2608 | 122 | 98 | 0.8 | |
|  | Blank mean 2509 Blank s = 266 | | | |  |
|  |  |  |  |  | |
| crRNA-S01/A20T RNA |  | | | |  |
| Concentration | **Raw Fluorescence** |  | **Background subtracted intensity** | **I/σ(I)** | |
|  | **Mean** | **σ** | **Mean** |  | |
| 1 uM | 15129 | 162 | 14013 | 86.6 | |
| 100 nM | 15069 | 345 | 13952 | 40.5 | |
| 10 nM | 13574 | 2133 | 12458 | 5.8 | |
| 1 nM | 9623 | 1836 | 8507 | 4.6 | |
| 100 pM | 1794 | 390 | 678 | 1.7 | |
| 10 pM | 1217 | 151 | 101 | 0.7 | |
| 1 pM | 1198 | 187 | 82 | 0.4 | |
|  | Blank mean 1116 Blank s = 191 | | | |  |
|  |  |  |  |  | |
| crRNA-S01/wt *HBB* RNA |  | | | |  |
| Concentration | **Raw Fluorescence** |  | **Background subtracted intensity** | **I/σ(I)** | |
|  | **Mean** | **σ** | **Mean** |  | |
| 1 uM | 5959 | 492 | 4760 | 9.7 | |
| 100 nM | 6402 | 711 | 5204 | 7.3 | |
| 10 nM | 1725 | 233 | 527 | 2.3 | |
| 1 nM | 1197 | 169 | -1 | 0.0 | |
| 100 pM | 1074 | 253 | -124 | -0.5 | |
| 10 pM | 1216 | 134 | 18 | 0.1 | |
| 1 pM | 1406 | 460 | 208 | 0.5 | |
|  | Blank mean 1198 Blank s = 76 | | | |  |

*I/σ(I), mean of background subtracted intensity divided by standard deviation of this mean.*

**Table S6. Fluorescence signal for SNP detection in contrived samples.**

| crRNA-B01 |  | | | |  |
| --- | --- | --- | --- | --- | --- |
| Targets | **Raw fluorescence** |  | **Background subtracted intensity** | **I/ σ(I)** | |
|  | **Mean** | **σ** | **Mean** |  | |
| HBB | 13314 | 293 | 11585 | 39.5 | |
| HBB / A20T | 11645 | 131 | 9916 | 75.6 | |
| A20T | 4292 | 566 | 2564 | 4.5 | |
|  | Blank mean 1729 Blank s = 143 | | | |  |
|  |  |  |  |  | |
| crRNA-S01 |  | | | |  |
| Targets | **Raw fluorescence** |  | **Background subtracted intensity** | **I/ σ(I)** | |
|  | **Mean** | **σ** | **Mean** |  | |
| HBB | 3702 | 451 | 174 | 0.4 | |
| HBB / A20T | 12817 | 360 | 9288 | 25.8 | |
| A20T | 12010 | 88 | 8481 | 96.7 | |
|  | Blank mean 3529 Blank s = 414 | | | |  |

*I/σ(I), mean of background subtracted intensity divided by standard deviation of this mean*
